# Haemophilus ducreyi Infection Induces Oxidative Stress, Central Metabolic Changes, and a Mixed Pro- and Anti-inflammatory Environment in the Human Host

**DOI:** 10.1128/mbio.03125-22

**Published:** 2022-12-01

**Authors:** Julie A. Brothwell, Kate R. Fortney, Hongyu Gao, Landon S. Wilson, Caroline F. Andrews, Tuan M. Tran, Xin Hu, Teresa A. Batteiger, Stephen Barnes, Yunlong Liu, Stanley M. Spinola

**Affiliations:** a Department of Microbiology and Immunology, Indiana University School of Medicine, Indianapolis, Indiana, USA; b Department of Medical and Molecular Genetics, Indiana University School of Medicine, Indianapolis, Indiana, USA; c Department of Pharmacology and Toxicology, University of Alabama at Birmingham, Birmingham, Alabama, USA; d Department of Medicine, Indiana University School of Medicine, Indianapolis, Indiana, USA; e Ryan White Center for Pediatric Infectious Diseases and Global Health, Department of Pediatrics, Indiana University School of Medicine, Indianapolis, Indiana, USA; f Division of Pulmonary, Allergy, and Critical Care, Emory University, Atlanta, Georgia, USA; g Targeted Metabolomics and Proteomics Laboratory, University of Alabama at Birmingham, Birmingham, Alabama, USA; h Department of Biostatistics, Indiana University School of Medicine, Indianapolis, Indiana, USA; i Department of Pathology and Laboratory Medicine, Indiana University School of Medicine, Indianapolis, Indiana, USA; McGovern Medical School

**Keywords:** *Haemophilus ducreyi*, dual RNA-seq, metabolome, interactome, human

## Abstract

Few studies have investigated host-bacterial interactions at sites of infection in humans using transcriptomics and metabolomics. Haemophilus ducreyi causes cutaneous ulcers in children and the genital ulcer disease chancroid in adults. We developed a human challenge model in which healthy adult volunteers are infected with H. ducreyi on the upper arm until they develop pustules. Here, we characterized host-pathogen interactions in pustules using transcriptomics and metabolomics and examined interactions between the host transcriptome and metabolome using integrated omics. In a previous pilot study, we determined the human and H. ducreyi transcriptomes and the metabolome of pustule and wounded sites of 4 volunteers (B. Griesenauer, T. M. Tran, K. R. Fortney, D. M. Janowicz, et al., mBio 10:e01193-19, 2019, https://doi.org/10.1128/mBio.01193-19). While we could form provisional transcriptional networks between the host and H. ducreyi, the study was underpowered to integrate the metabolome with the host transcriptome. To better define and integrate the transcriptomes and metabolome, we used samples from both the pilot study (*n* = 4) and new volunteers (*n* = 8) to identify 5,495 human differentially expressed genes (DEGs), 123 H. ducreyi DEGs, 205 differentially abundant positive ions, and 198 differentially abundant negative ions. We identified 42 positively correlated and 29 negatively correlated human-H. ducreyi transcriptome clusters. In addition, we defined human transcriptome-metabolome networks consisting of 9 total clusters, which highlighted changes in fatty acid metabolism and mitigation of oxidative damage. Taken together, the data suggest a mixed pro- and anti-inflammatory environment and rewired central metabolism in the host that provides a hostile, nutrient-limited environment for H. ducreyi.

## INTRODUCTION

Few studies have investigated infected environments from the perspective of both the human host and pathogen (1, 2). Haemophilus ducreyi is a Gram-negative extracellular pathogen that causes cutaneous skin ulcers on the limbs of children in the tropics and the sexually transmitted disease chancroid in adults (3, 4). To study interactions between H. ducreyi and the human host, we developed a human challenge model in which healthy adult volunteers are infected with H. ducreyi on the upper arm until they develop pustules (5).

In the human challenge model, H. ducreyi enters the host through breaks in the skin (6). The host mounts an immune response to the invading bacteria, and papules form within ~24 h. Neutrophils and macrophages attempt to phagocytose the bacteria to clear the infection; however, most experimentally infected volunteers fail to clear the infection and the papules evolve into pustules (7, 8). In pustules, H. ducreyi remains extracellular by secreting the antiphagocytic effectors LspA1 and LspA2 (9). For safety reasons, experimental infection is only allowed to progress to the pustular stage of disease, usually for 6 to 8 days after inoculation. During this time, neutrophils, macrophages, dendritic cells (DCs), T cells, and NK cells are recruited to the site of infection (8, 10–13). The architecture of experimental lesions resembles a suppurative granuloma in which macrophages admixed with regulatory T cells (Tregs) form a collar below the neutrophilic abscess, while memory T cells and NK cells remain below the collar (8). This architecture is likely due to induction of indoleamine 2,3-dioxygenase in DCs by H. ducreyi (14), which promotes expansion of the regulatory T cells in the collar and depletes tryptophan, which is required for memory T cell survival (15–17).

Interactions between H. ducreyi and the human host have been primarily explored in mutant versus parent trials in which volunteers are infected with an isogenic mutant on one arm and the parent strain on the other arm (18). In addition to antiphagocytic effectors LspA1 and LspA2 (9), essential virulence factors defined by these trials include those that prevent complement deposition (DsrA and DltA) (19–22), take up hemoglobin (HgbA) (23, 24), form microcolonies (proteins encoded by the *flp-tad* operon) (25–27), bind to collagen (NcaA) or fibrinogen (FgbA) (28, 29), or resist killing by antimicrobial peptides (SapA, SapB, and SapC) (30).

Although mutant versus parent trials have identified important host bacterial interactions, testing the phenotype of individual mutants does not give a comprehensive view of these interactions. We recently applied network analyses to identify global transcriptional interactions between H. ducreyi and the human host (31). In a pilot study, we generated a preliminary transcriptional interaction network between H. ducreyi and the human host using infected and wounded site samples from 4 volunteers (31). We also determined the metabolomes from infected and wounded site samples obtained from 4 volunteers, 3 of whom provided samples for the transcriptome data sets. However, the number of samples was insufficient to generate a transcriptome-metabolome interaction network.

Using the data from the pilot study, we calculated that samples from 12 pustule formers would have an 80% power to detect differentially expressed (DE) H. ducreyi gene transcripts at a fold change of >2 (infected site versus the inoculum) and a 90% power to detect DE human gene transcripts at a fold change of >2 (infected site versus a wounded site). To better define host-bacterial interactions in the abscess environment, we used the 4 paired transcriptional data sets from the previous study (31) and collected paired infected and wounded site samples from 8 additional volunteers, determined the transcriptomes of both the bacteria and the host using dual transcriptome sequencing (RNA-seq), and identified the metabolites present in the skin samples. We also examined interactions between the host transcriptome and metabolome using network analyses.

## RESULTS

### Human volunteers and tissue specimens.

Biopsy samples from pustules and wounded sites were collected from 12 adults (7 men and 5 women; 1 Black, 2 Hispanic, 1 mixed race, 8 White; mean age ± standard deviation [SD], 38.8 ± 14.9 years), 4 of whom participated in the pilot study and 8 of whom were specifically infected for this study (see Table S1 in the supplemental material) (31). The 12 volunteers were inoculated on their upper arm with a mean ± SD estimated delivered dose (EDD) of 95.1 ± 36.6 CFU of H. ducreyi strain 35000HP at three sites and with phosphate-buffered saline (PBS) (Table 1). The volunteers were infected until they developed a painful pustule 6 to 8 days after inoculation, at which point at least one infected site and the wounded site was biopsied with a 6-mm punch forceps. Either whole or half biopsy specimens from pustules and wounded sites were used for transcriptomic or metabolomic analysis.

**TABLE 1:** Number of high-quality reads from each transcriptome

Subject no.	Sample type	Raw reads, millions	High-quality reads^*a*^			
			*H. ducreyi*		Human	
			Millions	%	Millions	%
469	Wound	129.6	NA^*b*^	NA	106.1	81.9
	Pustule	590.5	0.172	0.0291	471.4	79.8
470	Wound	141.6	NA	NA	117.5	83.0
	Pustule	653.2	0.024	0.0037	514.6	78.8
471	Wound	134.6	NA	NA	108.0	80.2
	Pustule	580.3	0.091	0.0157	461.8	79.6
473	Wound	111.0	NA	NA	92.1	83.0
	Pustule	586.2	0.131	0.0223	472.8	80.7
474	Wound	134.1	NA	NA	105.1	78.4
	Pustule	641.2	0.042	0.0066	517.8	80.8
475	Wound	136.3	NA	NA	113.8	83.5
	Pustule	545.3	0.291	0.0534	423.4	77.6
476	Wound	130.5	NA	NA	103.1	79.0
	Pustule	585.7	0.014	0.0024	471.2	80.5
477	Wound	114.4	NA	NA	94.2	82.3
	Pustule	567.2	0.002	0.0003	442.9	78.1

a High-quality concordant, unambiguous paired-end reads.

b NA, not applicable.

10.1128/mbio.03125-22.4 TABLE S1 Human volunteers and response to inoculation with H. ducreyi 35000HP. Download Table S1, XLSX file, 0.01 MB. Copyright © 2022 Brothwell et al. 2022 Brothwell et al. https://creativecommons.org/licenses/by/4.0/ This content is distributed under the terms of the Creative Commons Attribution 4.0 International license.

### Dual RNA-seq of biopsy specimens yields bacterial and human transcripts.

RNA was extracted from the wound and pustule samples and the 4 bacterial cultures used to inoculate the 8 volunteers. As detailed in the Materials and Methods, ribosomal RNAs were depleted, total RNA libraries were prepared, and paired-end 150-bp sequences were obtained. There was insufficient RNA remaining from the volunteers in the pilot study to repeat sequencing; we reanalyzed the reads obtained from the 4 pairs of biopsy specimens and the 4 bacterial cultures used to inoculate those volunteers along with the new sets of reads.

Reads from the 12 volunteers and 8 bacterial cultures were aligned against the human and H. ducreyi genomes. The number of reads obtained from each sample is shown in Table 1. Due to the cellular infiltrate, pustules typically yield more RNA than wounds. In addition, pustules contain ~1.8 × 10^5^ CFU of H. ducreyi (32), while the inocula contain ~1.0 × 10^8^ CFU (5). To mitigate differences in gene expression due to sequencing depth, as opposed to differential expression, human reads in pustules were subsampled to levels comparable to their corresponding wound sample. Similarly, the reads from the bacterial cultures were subsampled to be more comparable to the bacterial reads in the pustules. The counts from these samples were used for downstream differential gene expression analysis in DESeq2.

**FIG 1:**
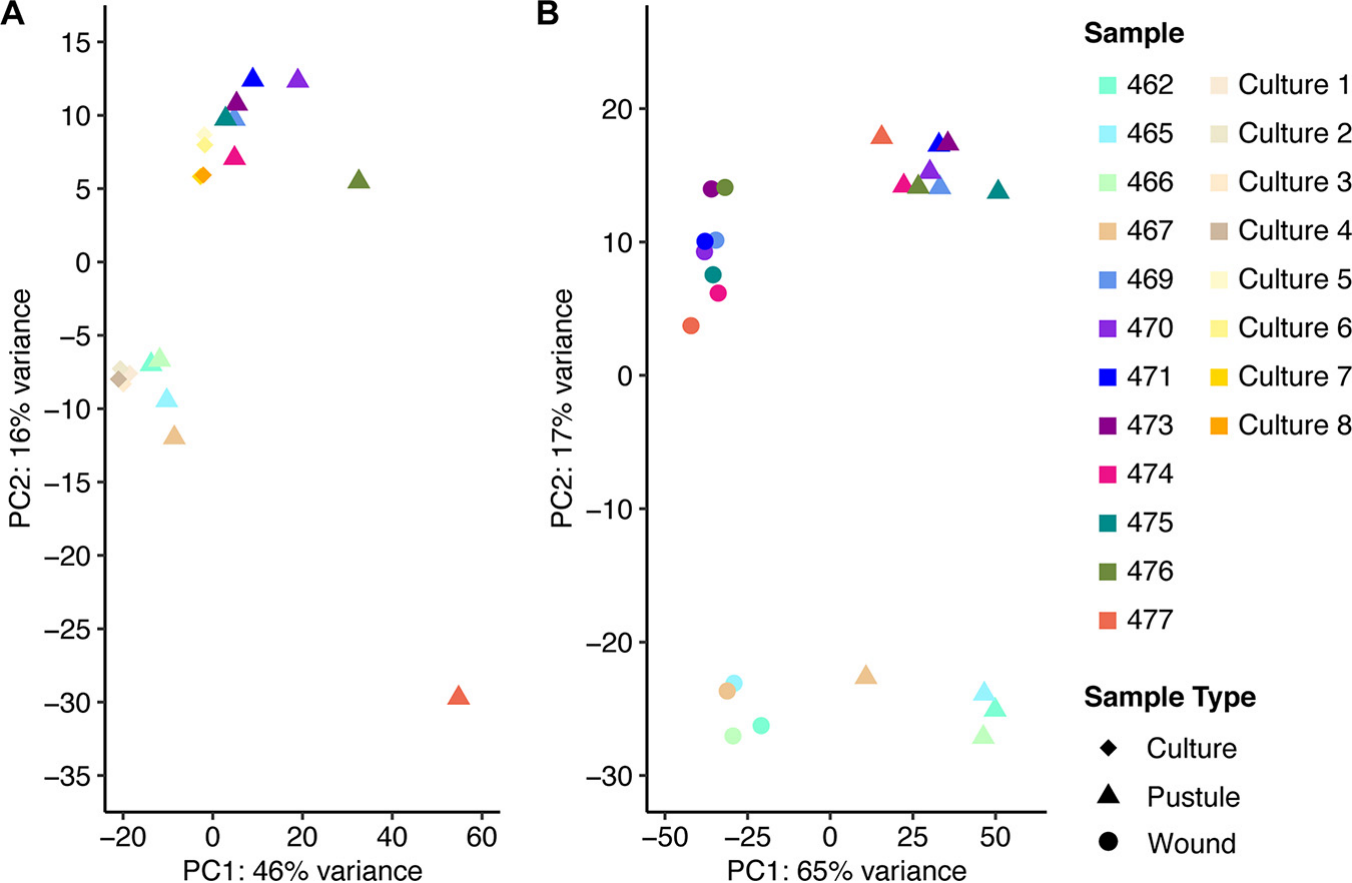
Principal-component analysis (PCA) of the human transcriptomes in pustule and wound samples (A) and between the inocula and H. ducreyi in pustules (B). Colors indicate samples corresponding to each volunteer or the inocula used to infect the volunteer, while the symbols correspond to the sample types. Note that some of the symbols for cultures 1 to 4 and cultures 5 to 8 are superimposed on each other.

By principal-component analysis (PCA), the transcriptomes of pustules were compared to their respective control transcriptomes (either the wound for human reads or the inoculum for H. ducreyi reads) (Fig. 1). We observed an apparent batch effect between the pilot samples and the new samples for both the human and H. ducreyi transcriptomes. Attempts to correct for the batch effect showed negligible improvement (data not shown). However, since each volunteer had a matched infected sample and wounded site sample, we performed a paired analysis to account for within-individual variation.

For the human transcriptomes, principal component 1 separated the pustule from wound samples and accounted for 65% of the variation between samples. Principal component 2 accounted for 17% of the variability and separated the pilot samples from the new samples; these differences likely arose from differences in the library preparation and/or sequencing platform. The transcriptomes of pustules were significantly different from those of wounds by permutational multivariate analysis of variance (PERMANOVA) (*P* = 0.001). All human samples had Pearson correlation coefficients of ≥0.86 (Fig. S1). For the bacterial transcriptomes, we saw a similar separation of the *in vivo* versus inoculum reads. The *in vivo* versus inoculum transcriptomes were significantly different by PERMANOVA (*P* = 0.008). Most of the *in vivo* H. ducreyi transcriptomes had Pearson correlation coefficients above 0.8; however, three pustule samples (470, 476, and 477) with lower numbers of bacterial reads had correlations in the range of 0.4 to 0.7.

10.1128/mbio.03125-22.2 FIG S1 Clustered heat maps of the Pearson correlation coefficients for all transcriptome samples. (A) Correlations between human transcriptomes in pustule and wound samples; (B) correlations between mid-log-phase cultures and pustule samples for H. ducreyi transcriptomes. Download FIG S1, TIF file, 1.9 MB. Copyright © 2022 Brothwell et al. 2022 Brothwell et al. https://creativecommons.org/licenses/by/4.0/ This content is distributed under the terms of the Creative Commons Attribution 4.0 International license.

### Differential expression of H. ducreyi genes *in vivo*.

**FIG 2:**
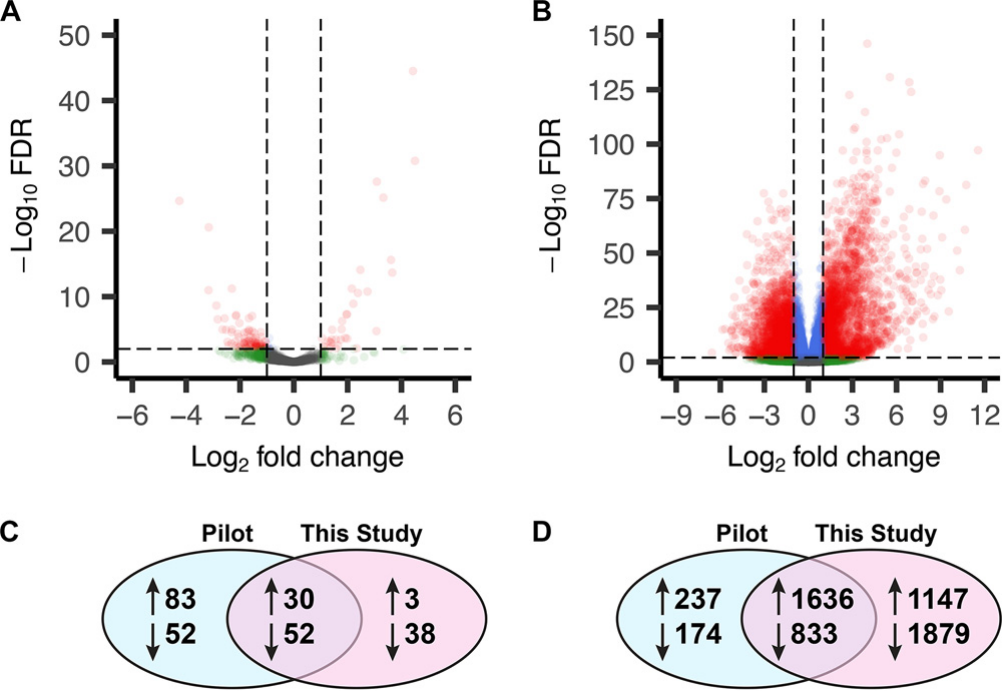
Volcano plots of the gene expression profiles for H. ducreyi (A) and human (B) DEGs. DEGs are shown in red. Genes meeting only the fold change or statistical significance cutoffs are shown in green and blue, respectively. Genes that exhibited no differences are in gray. Levels of expression of individual genes are shown in Table S1 and Table S2 for H. ducreyi and human genes, respectively. (C and D) DEGs that overlap in the H. ducreyi (C) and human (D) transcriptomes of this (*n* = 12) and the pilot (*n* = 4) study.

We next determined the differentially expressed genes (DEGs) between H. ducreyi in pustules and the inocula. We defined DEGs as having an absolute log_2_ fold change of ≥1 and a false-discovery rate (FDR) of <0.01. Using these criteria, we identified 33 upregulated H. ducreyi genes and 90 downregulated genes in pustules compared to the inocula (Fig. 2A and Table S2). Of these, 30 upregulated DEGs and 52 downregulated DEGs overlapped with those identified in our pilot study (Fig. 2C) (31); the overlap was significant for both upregulated (*P* = 1.1 × 10^−34^) and downregulated (*P* = 2.9 × 10^−46^) DEGs.

10.1128/mbio.03125-22.5 TABLE S2 H. ducreyi culture versus pustule DEGs. Download Table S2, XLSX file, 0.03 MB. Copyright © 2022 Brothwell et al. 2022 Brothwell et al. https://creativecommons.org/licenses/by/4.0/ This content is distributed under the terms of the Creative Commons Attribution 4.0 International license.

The H. ducreyi DEGs were classified using KEGG terms (Table 2) (33). The downregulated DEGs were mostly genes related to shifts in metabolism, including many nutrient transporter genes. There was also a large proportion of hypothetical genes (20/90) that were downregulated. The most-upregulated set of DEGs were in the *flp-tad* locus, which is required for the formation of bacterial microcolonies and for pustule formation in human volunteers (26, 27). In addition to the *flp-tad* operon, 17 genes or operons are required for the full expression of virulence of 35000HP in humans based on mutant versus parent trials (18): of these, three genes that encode proteins that are antiphagocytic effectors (*lspB-lspA2*) (34), take up hemoglobin (*hgbA*) (35), and confer serum resistance (*dsrA*) (20), were upregulated. However, three genes (*cpxA*, *hfq*, and *pal*) that are also required for virulence were downregulated (36–38). Of 15 genes or operons not required for virulence, two (*ompP2A* and *hhdB*) were upregulated and 5 (*ftpA*, *cdtC*, *momp*, *ompP2B*, and *cpxR*) were downregulated (Table S2) (24, 39–42). Although the differential expression of genes does not appear to correlate with their requirement for virulence, the data suggest that adjustments in metabolism, the formation of microcolonies, hemoglobin uptake, and resistance to phagocytosis and complement-mediated killing are essential for H. ducreyi survival in the environment of an abscess. The downregulation of genes involved in nucleotide, amino acid, glycan, and cofactor metabolism may reflect their low availability in the environment of a pustule or less robust growth *in vivo* than in mid-log-phase cultures.

**TABLE 2:** Bacterial DEG KEGG classifications

Category	No. of DEGs:	
	Upregulated	Downregulated
Metabolism		
Carbohydrate metabolism	3	4
Energy metabolism	1	1
Lipid metabolism	0	1
Nucleotide metabolism	0	5
Amino acid metabolism	0	3
Metabolism of other amino acids	0	3
Glycan biosynthesis and metabolism	0	4
Metabolism of cofactors and vitamins	0	4
Metabolism of terpenoids and polyketides	0	1
Other	2	3
Total	6	29
Genetic information processing		
Transcription	2	0
Translation	1	3
Folding, sorting, and degradation	2	7
Replication and repair	1	1
Total	6	11
Environmental information processing		
Membrane transport	4	14
Signal transduction	0	3
Signaling molecules and interaction	12	2
Total	16	19
Human diseases		
Drug resistance—antimicrobial	0	2
Total	0	2
Miscellaneous		
Putative function	4	9
No known function	1	20
Total	5	29
Total	33	90

### Differential expression of human genes in pustules.

**FIG 3:**
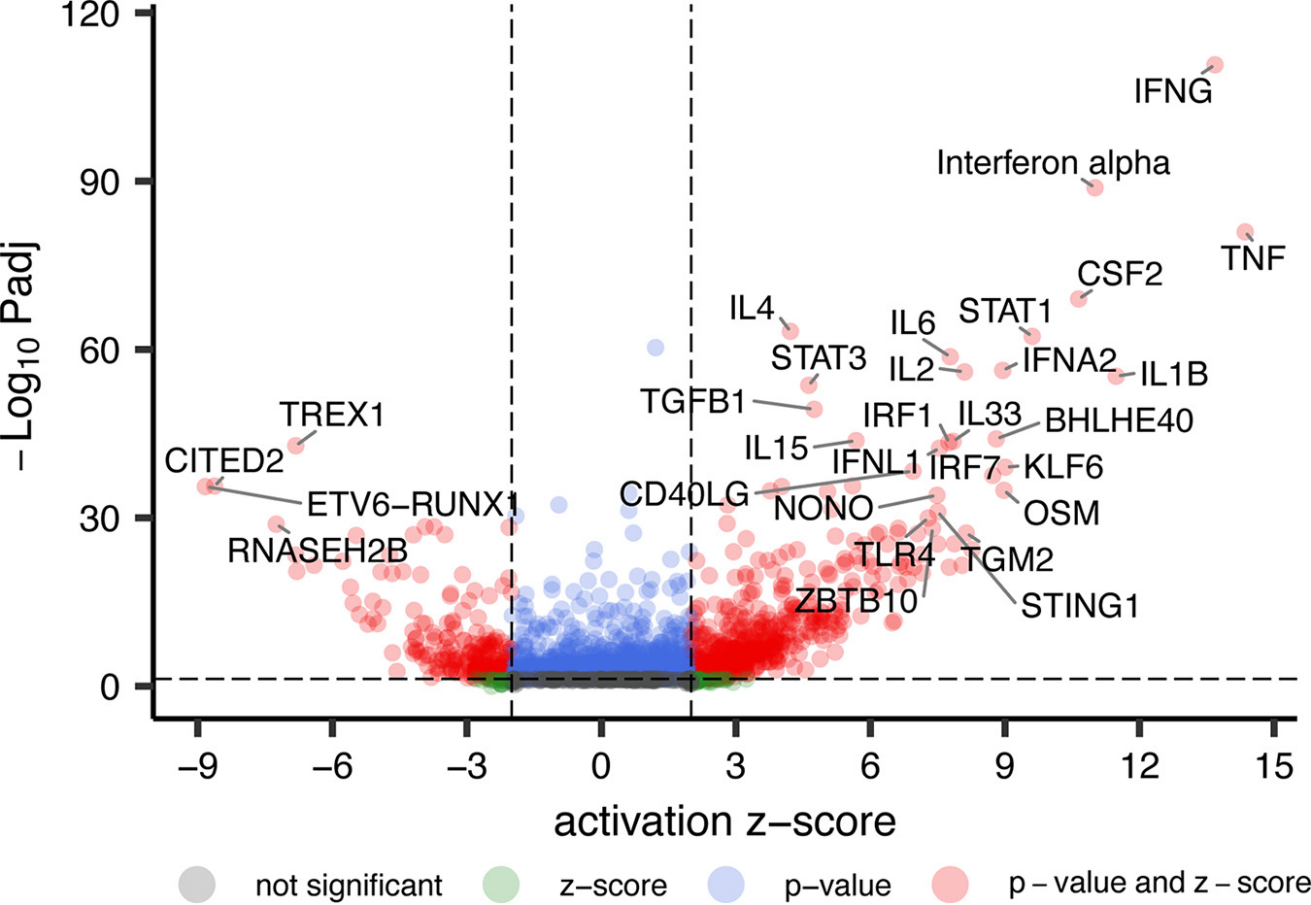
Volcano plot of pathways predicted to be activated in pustules by Ingenuity Pathway Analysis. Predictions are based off the top 2,000 human DEGs. The top 30 identified pathways corresponding to human genes and protein products are labeled on the plot. Significance was defined as a 2-fold difference in activation Z-score and *P* < 0.05.

We also determined the human DEGs, defined as having an absolute log_2_ fold change of ≥1 and a false-discovery rate (FDR) of <0.01, in pustules compared to wounded skin. These cutoffs identified 2,783 upregulated and 2,712 downregulated DEGs (Fig. 2B and Table S3). Of these, 1,636 upregulated DEGs and 833 downregulated DEGs overlapped with those identified in our pilot study (Fig. 2D) (31); the overlap was significant for both sets (*P* < 0.00001). Human DEGs were classified using gene set enrichment analysis (GSEA) to define the top 20 altered biological pathways in the up- and downregulated gene lists (43). The most-upregulated pathways in pustules indicated that there was a robust immune infiltrate that included activation of leukocytes (Table 3). The most-downregulated pathways in pustules indicated a disruption in cell-cell adhesion and signaling as well as a downregulation of lipid metabolism (Table 4). There are also several downregulated biological processes relating to neurons, suggesting that the bacterial infection and/or the immune infiltrate damaged peripheral nerve fibers. Ingenuity Pathway Analysis (IPA) predicted upregulated signaling primarily through type I and II interferons as well as the presence of several interleukins and Toll-like receptor (TLR) activation (Fig. 3).

**TABLE 3:** GO pathway analysis of upregulated human DEGs in pustules versus wounds

GO biological process term	NES^*a*^	FDR
Cell activation	10.61	<0.0001
Defense response to other organism(s)	10.21	<0.0001
Positive regulation of the immune system	9.91	<0.0001
Regulation of immune response	9.74	<0.0001
Lymphocyte activation	9.61	<0.0001
Innate immune response	9.49	<0.0001
Immune effector process	9.19	<0.0001
Positive regulation of immune response	8.65	<0.0001
Regulation of cell activation	8.64	<0.0001
Inflammatory response	8.62	<0.0001
T cell activation	8.61	<0.0001
Adaptive immune response	8.58	<0.0001
Cytokine production	8.42	<0.0001
Regulation of lymphocyte activation	8.32	<0.0001
Leukocyte-mediated immunity	8.01	<0.0001
Response to bacterium	7.89	<0.0001
Leukocyte cell-cell adhesion	7.75	<0.0001
Positive regulation of cell activation	7.72	<0.0001
Regulation of immune effector process	7.66	<0.0001
Leukocyte differentiation	7.61	<0.0001

a NES, normalized enrichment score.

**TABLE 4:** GO pathway analysis of downregulated human DEGs in pustules versus wounds

GO biological process term	NES^*a*^	FDR
Homophilic cell adhesion via plasma membrane adhesion molecules	−4.09	<0.0001
Cell-cell adhesion via plasma membrane adhesion molecules	−3.65	<0.0001
Fatty acid metabolic process	−3.60	<0.0001
Cellular lipid metabolic process	−3.50	<0.0001
Lipid metabolic process	−3.36	<0.0001
Neuron differentiation	−3.32	<0.0001
Synapse assembly	−3.23	<0.0001
Lipid biosynthetic process	−3.18	<0.0001
Organic acid metabolic process	−3.06	2.53E−04
Monocarboxylic acid and catabolic process	−3.02	2.73E−04
Neuron development	−2.96	3.95E−04
Organic hydroxy compound metabolic process	−2.92	3.84E−04
Glutamate receptor signaling pathway	−2.89	4.70E−04
Cell junction assembly	−2.86	5.14E−04
Sensory organ development	−2.84	7.28E−04
Monocarboxylic acid metabolic process	−2.81	9.05E−04
Regulation of synapse assembly	−2.81	9.57E−04
Cell surface receptor signaling pathway involved in cell-cell signaling	−2.76	1.07E−03
Neurogenesis	−2.75	1.11E−03
Olefinic compound metabolic process	−2.72	1.48E−03

a NES, normalized enrichment score.

10.1128/mbio.03125-22.6 TABLE S3 Human wound versus pustule DEGs. Download Table S3, XLSX file, 0.6 MB. Copyright © 2022 Brothwell et al. 2022 Brothwell et al. https://creativecommons.org/licenses/by/4.0/ This content is distributed under the terms of the Creative Commons Attribution 4.0 International license.

### Decreases in bacterial cellular metabolism are associated with leukocyte influx.

**FIG 4:**
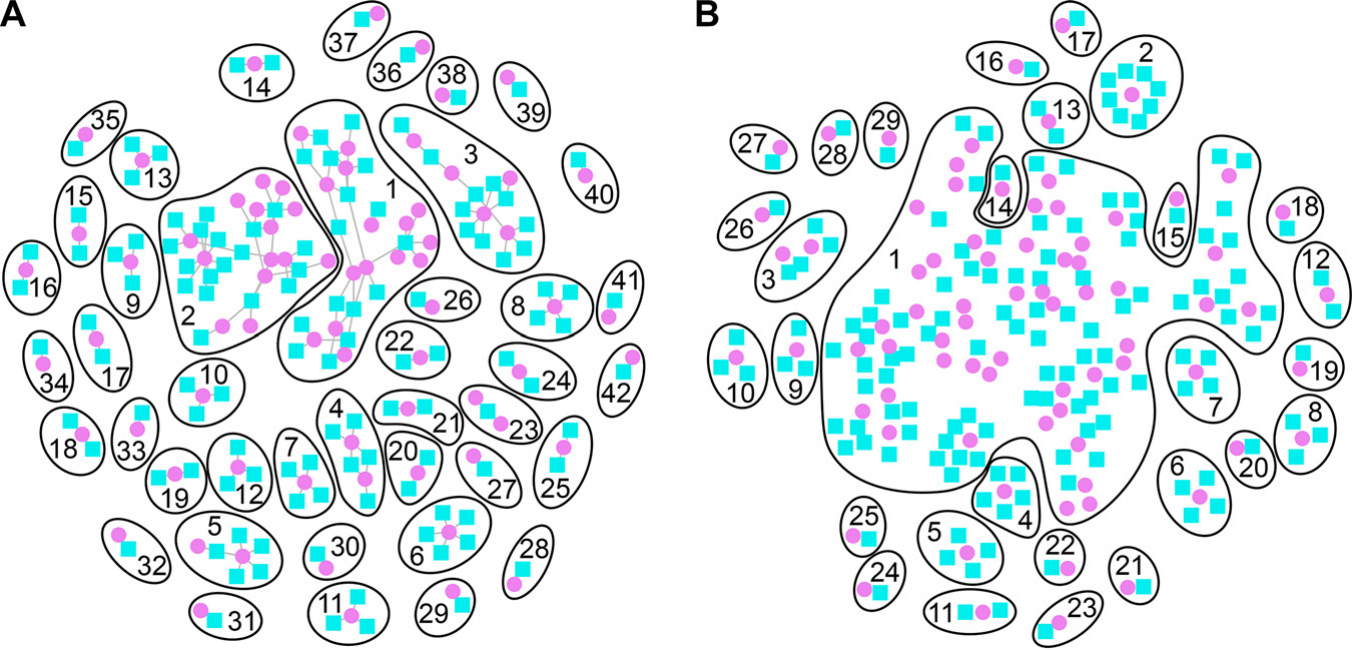
Bipartite network graphs of H. ducreyi genes (magenta circles) whose abundance was positively correlated with the abundance of human genes (cyan squares) (A) and whose abundance was negatively correlated (B) at the level of |*r*| > 0.75 and *P* < 0.005 are shown. The genes belonging to each cluster are shown in Table S4.

To define significant correlations between H. ducreyi and human DEGs, we normalized reads for both the human and H. ducreyi data by variance-stabilizing transformation (vst) and generated bipartite networks to identify genes whose expression is correlated independent of whether the genes are up- or downregulated in samples overall. We identified human and H. ducreyi DEGs that either simultaneously increased or decreased (positive correlations) or where an increase in one DEG corresponded to a decrease in another (negative correlations). Clusters were generated based on DEGs that had an |*r*| value of >0.75 and a *P* value of <0.005. We identified 42 positively associated clusters and 29 negatively associated clusters (Fig. 4; Table S4). We highlight insights from some of the clusters below.

10.1128/mbio.03125-22.7 TABLE S4 Bipartite network genes. (A) Positive correlations; (B) negative correlations. Download Table S4, XLSX file, 0.03 MB. Copyright © 2022 Brothwell et al. 2022 Brothwell et al. https://creativecommons.org/licenses/by/4.0/ This content is distributed under the terms of the Creative Commons Attribution 4.0 International license.

Although bacterial differential expression analysis indicated decreased bacterial growth in pustules, the largest cluster of positively correlated genes (Fig. 4A, cluster 1) predicted that increases in bacterial adhesion/microcolony formation (*flp-tad* locus), bacterial cell growth via tRNA modification (*trmA*), the pentose phosphate pathway (PPP) (*gnd*), arginine biosynthesis (*argG*), mannose metabolism (*manB*), and peptidoglycan synthesis (*ampD*) were correlated with the increased presence of polymorphonuclear leukocytes (PMNs) attempting phagocytosis (*CXCL6*, *NLRP6*, and *UNC119*) and apoptosis (*RELL2*) versus cell proliferation (*PAX3*) in the pustule. The bacterial DEGs in positively correlated cluster 2—which are all downregulated in pustules by differential expression analysis—also indicated that bacterial cell division (*zapA*, *lppC*, and *HD_0257*), protein secretion (*secB*), and stabilized (p)ppGpp levels (*mazG*) were correlated with increases in host Toll-like receptor (TLR) activation and subsequent NF-κB and type I interferon signaling (*IRF7*, *LY96*, and *TLR6*). These clusters may be positively correlated with host TLR signaling because H. ducreyi expresses lipooligosaccharide (LOS), the equivalent of lipopolysaccharide (LPS), and increased growth would increase the absolute abundance of LOS (44). TLR4 (which is upregulated in pustules, but not part of the cluster) in association with LY96 can sense LPS (45). Another gene in cluster 2, *PPP1R16B*, is also upregulated and protects endothelial cells from LPS-induced leakage (46). Increases in bacterial methionine import (*metN*) and proteolysis (*hslV*) (cluster 4), also correlated with activation of TLR4 (*RPS6KA4*). Taken together, the bacterial genes in clusters 1 and 2 may drive the activation of the top upstream regulators predicted by IPA (Fig. 3).

Our transcriptional data suggest that H. ducreyi struggles with nucleotide metabolism, since it lacks multiple enzymes for *de novo* purine synthesis and the transcription of purine salvage pathway enzymes, including *apt* (cluster 3), is decreased. Host genes indicating damage of host tissue and host cell apoptosis also correlated with bacterial genes involved with growth in cluster 3. Taken together, the upregulation of *AEN* and *ANGPT2* (where *VEGFD* expression is decreased) suggests the presence of DNA damage and the loosening and death of endothelial cells (47, 48). While there are signs of cell proliferation/tissue repair (e.g., upregulation of *PCNA*), mitosis is likely stalled in these cells (*PKMYT1*).

Most bacterial genes in the positive correlation clusters 1 to 4 also appeared in negative correlation cluster 1. Fourteen of the 74 protein-coding host genes in negatively correlated cluster 1 function specifically in neurons, suggesting that nerve function and signaling are altered in H. ducreyi infection, which may explain why H. ducreyi ulcers are painful. In agreement with our GSEA results, most of these genes are significantly transcriptionally downregulated in pustules (Tables 3 and 4). Bacterial growth and adhesion are also negatively correlated with phagocytosis and actin rearrangement (*ARGHAP23*, *IQGAP2*, *ELMO1*, and *MRC1*), immune response (*C6*, *DOK3*, *GGT5*, *IL1R1*, *OSMR*, and *RXRA*), and altered cellular metabolism (*SLC16A11*, *STEAP4*, *SSC5D*, *ST3GAL1*, and *TIGAR*). These results are consistent with H. ducreyi growth and survival in lesions being dependent on microcolony formation and resistance to phagocytosis (9, 26).

### Metabolic differences in pustules compared to wounds.

**FIG 5:**
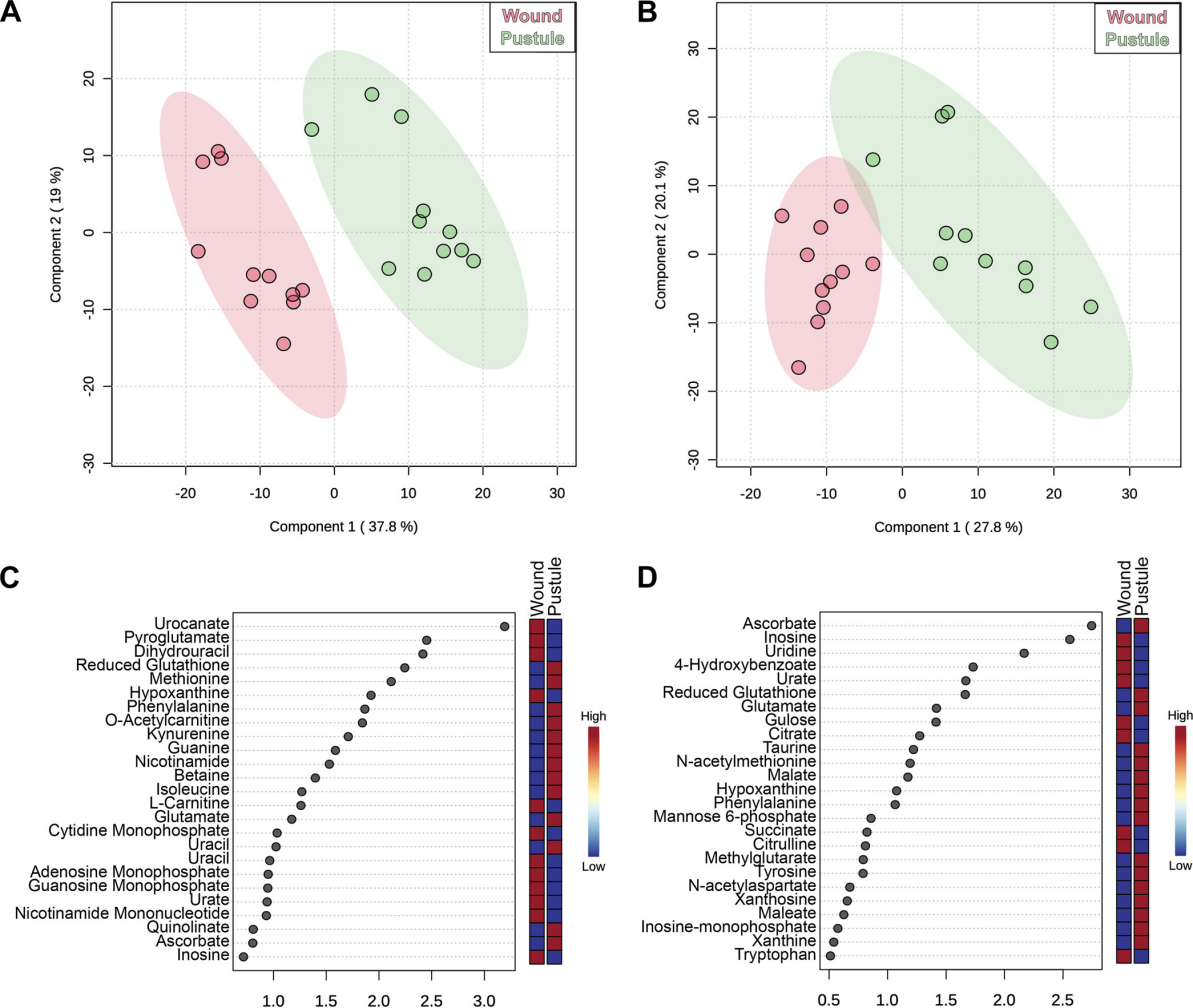
The variability between metabolome samples was assessed by partial least-squares discriminant analysis (PLS-DA) for positive ions (A) and negative ions (B). The most variable ions between each sample type are annotated in the variable importance of projection (VIP) plots for the positive (C) and negative (D) ions. Ions in the VIP plot were identified based on MS1 spectral annotations.

To further define the pustular microenvironment, we performed untargeted metabolomics by ultraperformance liquid chromatography-tandem mass spectrometry (UPLC-MS/MS) in both the positive- and negative-ion modes. As we had sufficient samples remaining from subjects 462, 465, and 466 in the pilot study (31), their samples were reanalyzed along with the new samples. Metabolites in the 11 paired samples were identified using spectra obtained in house of the IROA 600 standard compound library. We identified 432 positive and 381 negative ions, of which 205 and 198, respectively, had significant differential abundance in pustules versus wounds (Table S5 and Table S6). Clustering of metabolomes by partial least-squares (PLS) discriminant analysis revealed that the metabolomes of like sample types clustered together and that the wound and pustule metabolomes were distinct in both the positive- and negative-ion modes (Fig. 5A and B, respectively).

10.1128/mbio.03125-22.8 TABLE S5 Differentially abundant positive ions. Download Table S5, XLSX file, 0.07 MB. Copyright © 2022 Brothwell et al. 2022 Brothwell et al. https://creativecommons.org/licenses/by/4.0/ This content is distributed under the terms of the Creative Commons Attribution 4.0 International license.

10.1128/mbio.03125-22.9 TABLE S6 Differentially abundant negative ions. Download Table S6, XLSX file, 0.07 MB. Copyright © 2022 Brothwell et al. 2022 Brothwell et al. https://creativecommons.org/licenses/by/4.0/ This content is distributed under the terms of the Creative Commons Attribution 4.0 International license.

The ions driving the separations are shown in the variable importance of projection (VIP) plots for the top 25 ions (Fig. 5C and D). Consistent with the results of the pilot study (31), ascorbate was the main driver differentiating between the wound and pustule samples for the negative ions, and pustules had a higher abundance of ascorbate (Fig. 5).

Wounds were more abundant in nucleotides (inosine, uridine, CMP, AMP, and GMP) and tricarboxylic acid cycle (TCA) intermediates (citrate and succinate) than pustules. In wounds, energy is also likely produced through β-oxidation of fatty acids (l-carnitine) and synthesis of oxidizing equivalents (nicotinamide mononucleotide). Some ions, such as urate (purine salvage pathway), suggested that inflammation was present in wounds.

In contrast, pustules exhibited higher abundances of amino acids—several of which were *N-*acetylated—suggesting that protein degradation was occurring (49, 50). Pustules also contained higher abundances of the antioxidative metabolites glutathione and taurine, which could be increased to neutralize damage from the oxidizing pustular environment. Multiple components of the nucleotide salvage pathway were present (hypoxanthine, IMP, and xanthine) as well as the kynurenine pathway (kynurenine, quinolinate, and nicotinamide), suggesting that pustules contain metabolites supporting cellular replication and redox cofactors.

### Differentially active human metabolic pathways in pustules.

To determine which metabolic pathways were enriched in our samples, the MS1 spectra were analyzed by Mummichog (51). Mummichog further supported the differential abundance of metabolites in several amino acid metabolism pathways (tyrosine, alanine and aspartate, histidine, lysine, and tryptophan), nucleotide metabolism (purine, pyrimidine, and PPP), energy metabolism (glycolysis and gluconeogenesis, the TCA cycle, and nicotinate), neurotransmitter production (biopterin), and tissue protective metabolites (glutathione and β-alanine) (Tables 5 and 6).

**TABLE 5:** Enriched pathways in pustules for the positive-ion mode

Pathway	Pathway size	No. of hits	*P* value
Tyrosine metabolism	46	27	0.0131
Selenoamino acid metabolism	5	4	0.0172
Urea cycle/amino group metabolism	34	18	0.0213
Pyrimidine metabolism	25	13	0.0276
Alanine and aspartate metabolism	17	9	0.0305
Vitamin B_3_ (nicotinate and nicotinamide) metabolism	15	8	0.0314
β-Alanine metabolism	13	7	0.0324
Purine metabolism	26	13	0.0339
Vitamin H (biotin) metabolism	5	3	0.0378
Biopterin metabolism	5	3	0.0378
Pentose phosphate pathway	5	3	0.0378
Histidine metabolism	20	10	0.0379
Lysine metabolism	20	10	0.0379
Tryptophan metabolism	29	14	0.0406
Glutathione metabolism	12	6	0.0464
Drug metabolism—cytochrome P450	10	5	0.0498
Nitrogen metabolism	10	5	0.0498

**TABLE 6:** Enriched pathways in pustules for the negative-ion mode

Pathway	Pathway total	No. of hits	*P* value
Glycolysis and gluconeogenesis	8	6	0.0333
Drug metabolism—cytochrome P450	13	8	0.0368
TCA cycle	8	5	0.0439

**FIG 6:**
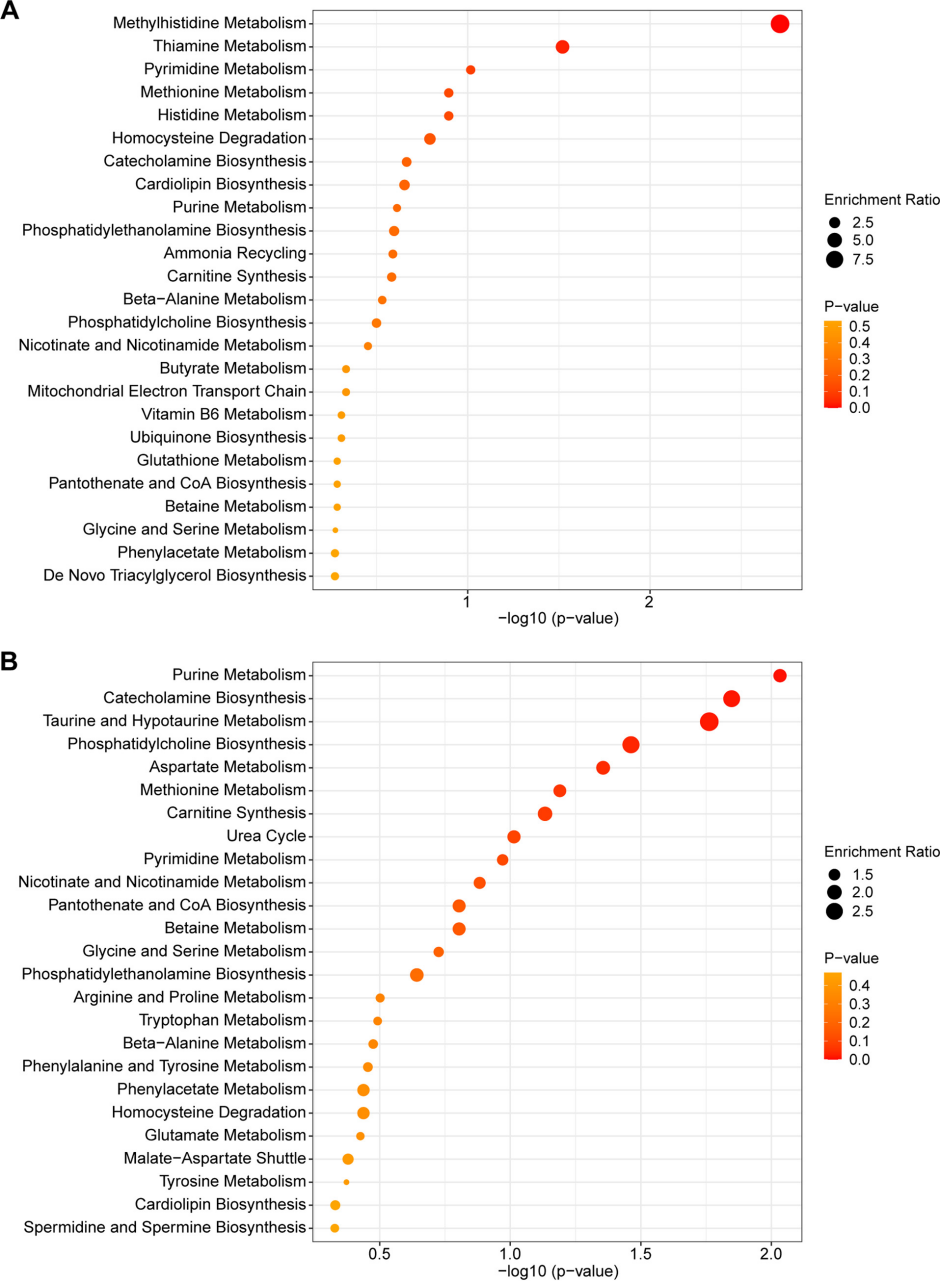
The top 25 enriched pathways by MSEA analysis in wounds (A) and pustules (B) are shown in the bubble plots. The size of the bubble corresponds to how enriched the pathway is compared to the data set, and the color corresponds to the statistical significance of the pathway.

We also used metabolite set enrichment analysis (MSEA) to identify enriched metabolic pathways in the wounds and pustules from ions that were differentially abundant (FDR < 0.05) (52). There were two significantly enriched pathways in wounds: methylhistidine metabolism (*P* = 0.002) and thiamine metabolism (*P* = 0.030) (Fig. 6A). There were three significantly enriched pathways in pustules—purine metabolism (*P* = 0.009), catecholamine biosynthesis (*P* = 0.014), and taurine and hypotaurine metabolism (*P* = 0.0173)—again emphasizing the importance of nucleotide salvage pathways, neurotransmitters, and antioxidants in pustules (Fig. 6B). Network representations of the pathways that were detected for wounds and pustules are shown in Fig. S2A and B, respectively.

10.1128/mbio.03125-22.3 FIG S2 Network representation of enriched metabolic pathways in wounds. (A) Related to Fig. 6A; (B) related to Fig. 6B. The larger the size of the node, the more enriched the pathway is. The redder the node, the more statistically significant the node is. Edges represent pathways with shared metabolites. Download FIG S2, TIF file, 2.6 MB. Copyright © 2022 Brothwell et al. 2022 Brothwell et al. https://creativecommons.org/licenses/by/4.0/ This content is distributed under the terms of the Creative Commons Attribution 4.0 International license.

### Interaction network of metabolites and differentially expressed human genes.

**FIG 7:**
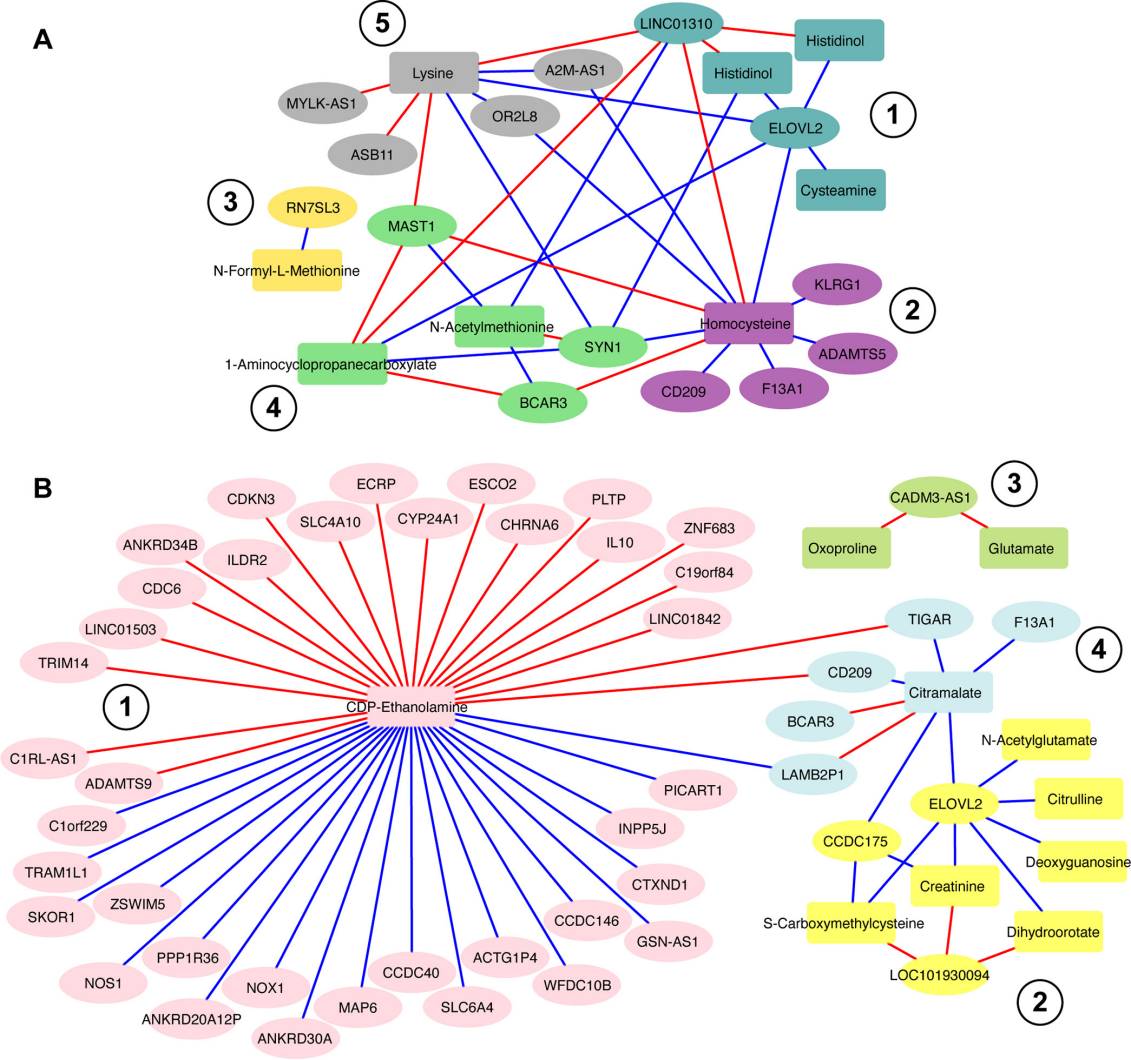
Metabolome and human transcriptome interaction networks between (A) the positive-ion metabolome and human transcriptome and (B) the negative-ion metabolome and human transcriptome. Clusters are numbered, and nodes in each cluster share the same color. Node shape indicates genes (ovals) and metabolites (rectangles). Edge color indicates positive (red) or negative (blue) correlations. Repeated ion names in the clusters reflect different ions of the same metabolite. Additional annotations of nodes are available in Table S7.

Given the overwhelming abundance of host cells relative to H. ducreyi in infected tissue, the metabolic environment of a pustule is likely driven primarily by the host response to infection. To explore whether there were novel linkages between the human transcriptome and the metabolites that were detected, we performed network analysis with xMWAS (53). We determined the correlation coefficients between the significant DEGs and either the negative or positive ions by PLS analysis and visualized the result for absolute correlation coefficients of >0.75 where *P* is <0.05. This yielded five human transcript-positive ion clusters and four human transcript-negative ion clusters (Fig. 7 and Table S7).

10.1128/mbio.03125-22.10 TABLE S7 Multiomic networks. (A) Positive ions versus the human transcriptome multiomic network; (B) negative ions versus the human transcriptome multiomic network. Download Table S7, XLSX file, 0.01 MB. Copyright © 2022 Brothwell et al. 2022 Brothwell et al. https://creativecommons.org/licenses/by/4.0/ This content is distributed under the terms of the Creative Commons Attribution 4.0 International license.

The networks highlighted potential links between fatty acid metabolism and oxidative damage in pustules. One of the most interconnected genes was *ELOVL2* (Fig. 7A, cluster 1, and Fig. 7B, cluster 2), which elongates very long polyunsaturated fatty acids and is upregulated in pustules. *ELOVL2* affects the ratio of M1 and M2 type macrophages and their phagocytic ability in mice (54). Macrophages from mice that lack *ELOVL2* exhibit a proinflammatory M1 phenotype, likely because several *ELOVL2* products mediate resolution of inflammation (55). *ELOVL2* was negatively correlated with positive-ion MS1 spectra corresponding to cysteamine, which is a breakdown product of pantothenate via coenzyme A (CoA) and pantethenine, and had a higher differential abundance in the pustule. Consistent with the higher abundance of cysteamine in pustules, vanin transcripts (*VNN1*, *VNN2*, and *VNN3*), which encode membrane pantetheinases, were upregulated in pustules. *VNN1* has been shown to skew macrophage differentiation toward an M2 type and promote granuloma formation in Coxiella burnetii infection (56). As H. ducreyi-infected sites contain M2 macrophages and resemble granulomas (57), *ELOVL2* may play a similar role in H. ducreyi infection. *ELOVL2* was negatively correlated with lysine, which in addition to roles in protein synthesis is used to produce carnitine to aid in fatty acid transport into and out of the mitochondria for β-oxidation (58). *ELOVL2* was also negatively correlated with deoxyguanosine and dihydroorotate (DNA replication) as well as citramalate, citrulline, creatinine, and *S*-carboxymethylcysteine, which have all been implicated as being anti-inflammatory in infection (59–61). Taken together, our data suggest that changes in fatty acid metabolism are correlated with regulation of inflammation and that *ELOVL2* may be a key regulator in this process.

CDP-ethanolamine has the greatest number of connections in the negative-ion clusters (Fig. 7B). CDP-ethanolamine is used to synthesize the lipid phosphoethanolamine. The increased abundance of CDP-ethanolamine was positively correlated with the upregulation of genes involved in DNA replication (*CDC6*, *ANKRD34B*, *CDKN3*, and *ESCO2*), epithelial barrier function (*ILDR2*), immune function and extracellular matrix (ECM) remodeling (*ADAMTS9*, *TRIM14*, *ECRP*, *IL-10*, *ZNF683*, and *CD209*), and phospholipid transfer (*PLTP*). CDP-ethanolamine was negatively correlated with the upregulation of genes for nitric oxide and proton transport (*NOS1* and *NOX1*, respectively), decreased microtubule stability (*MAP6* and *CCDC40*), decreased cell motility (*INPP5J*), and neuronal processes (*SKOR1* and *SLC6A4*). Together, these data suggest that higher abundance of CDP-ethanolamine is associated with cell growth and skin repair, whereas it is negatively associated with the immune response and neuronal function.

The network analysis also highlighted links to oxidative metabolism. For example, oxoproline and glutamate cluster (Fig. 7B, cluster 3); both metabolites are involved in glutathione synthesis. Additionally, *TIGAR* and *PICART1*, both p53-induced long noncoding RNAs (lncRNAs), were upregulated. Upregulation of TIGAR enhances glutathione production and helps to rewire central carbon metabolism from glycolysis to favor flux back through the oxidative PPP (62); upregulation of PICART1 enhances cell adhesion over cell migration (63). The data suggest that the immune infiltrate has optimized its metabolism to combat H. ducreyi infection, but the metabolic state may inhibit host cell migration, fostering the formation of a granuloma.

## DISCUSSION

To better understand the environment of H. ducreyi infection, we examined the metabolome and human and bacterial transcriptomes of infected and wounded sites. We subsequently performed both pathway and network analyses to examine molecular interactions during in infection. Our data expand upon a previous pilot study where we were able to generate provisional bipartite transcriptional networks. By increasing our sample size, the bipartite networks now have more robust “hub” genes, and we were also able to identify correlations between ion abundances from metabolites that correlated with human gene expression. The differences between the two studies may be due to differences in the RNA sequencing technology, software analysis and sample size, updates in human genome annotation files, and grouped versus paired analysis. Nevertheless, there was significant overlap in both the H. ducreyi and the human transcriptomes between this and the pilot studies.

We identified several genes and metabolites that suggest that the activation of innate immune cells leads to an oxidative environment and that the host attempts to mitigate the oxidative damage by increases in taurine, glutathione, and *S*-carboxymethylcysteine. We identified several pathways that tie the immune response to metabolism. For example, interferon gamma (*IFNG*) transcripts were upregulated along with indoleamine 2,3-dioxygenase (*IDO1/2*) transcripts and several downstream enzymes of the kynurenine pathway (64). This is consistent with the observation that H. ducreyi activates TLR4 on DCs via LOS resulting in IDO expression and metabolism of tryptophan to kynurenine (14). DCs from pustule formers display upregulated interleukin-10 (IL-10) secretion, potentially fostering the enrichment of Tregs observed in pustules (14, 16). Expression of both *IDO1/2* and *IL-10* appears in our study (see Table S3 in the supplemental material), and elements downstream of their activity appeared in our network analyses. Upregulated transforming growth factor β (TGF-β) is associated with the presence of regulatory DCs (DCregs) in pustule formers compared to resolvers (65) and increased Tregs in pustules (66). Consistent with these observations, *TGFB1* is upregulated in pustules (Table S3) and is predicted to be an important upstream regulator based on our transcriptomics results (Fig. 3). Immune suppression via DCregs and Tregs may promote pathogen survival but limit tissue damage as the infection progresses to the ulcerative stage (65, 66).

Compared to M1 macrophages, M2 macrophages are better able to phagocytose H. ducreyi (57); both types of macrophages are present in pustules, suggesting that the ratio between them may be key to infection outcome. Both pro- and anti-inflammatory gene transcripts and/or products are present in our data set. For example, the high abundance of citrulline suggests JAK2-STAT1 signaling is blocked, resulting in anti-inflammatory immune response (59). The upregulation of genes such as *ELOVL2*, which is required for inflammation resolution, also suggests an anti-inflammatory state (54, 55). In contrast, we also detected the presence of transcripts of proinflammatory cytokines such as *IL1B*, *IL-6*, and *CXCL8* (IL-8).

The increase in oxidant scavengers in pustules suggests that there is sufficient superoxide and/or free radicals to induce production of these protective metabolites. Optimal production of superoxide requires the rewiring of central carbon metabolism (67). Upregulation of *IDO1/2* could support the NAD^+^ synthesis required for production of oxidative metabolites through the *de novo* (i.e., kynurenine) pathway as well as the NAD^+^ salvage pathway, whose genes were moderately but not significantly upregulated (Table S3) (64, 67). Glycolysis followed by oxidative phosphorylation through the TCA cycle is optimal for ATP production and cell growth. However, the majority of cells recruited to infected sites (neutrophils and macrophages) are terminally differentiated and generate toxic compounds (e.g., superoxide), which requires a large pool of NAD(P)H to both drive oxide production and provide reducing equivalents to repair damage to the cell itself (68, 69). This is best accomplished by rerouting metabolic flux from glycolysis through the oxidative PPP (68). Although several transcripts encoding PPP enzymes only trended toward being upregulated in pustules, we did find significant upregulation of *TIGAR*, which is associated with promoting flux through the nonoxidative to oxidative PPP rather than glycolysis (62). Further evidence of PPP activity is the activation of the purine salvage pathway in human cells, which suggests that the nonoxidative branch of the PPP is active (Table 5 and Table S3). Since we used transcriptomics and a single time point for bulk metabolomics, we are limited in our interpretation of PPP activity.

Interestingly, our transcriptomics data predict that there is still flux through the latter half of glycolysis through to lactate production and export since transcripts for lactate dehydrogenase (*LDHA*) and the lactate transporter *SLC16A1* are significantly upregulated in pustules (Table S3). Lactate is higher in abundance in wound samples, which suggests that lactate is used as a carbon source in the pustule. Glycolysis may be used to deplete glucose from the environment and provide phagocytes with the requisite energy to mount the immune response (67). Addition of lactate to macrophages prior to Mycobacterium tuberculosis infection increased glycolysis and improved bacterial clearance despite reduced proinflammatory cytokine secretion (70). A similar paradigm may function for H. ducreyi, since M2 macrophages phagocytose H. ducreyi (57).

Our ability to identify metabolic pathways in H. ducreyi *in vivo* is limited. Although we compensated for the difference in the sizes of the human and H. ducreyi genomes and ratio of host to bacterial cells with higher read depth for pustule samples, coverage for the H. ducreyi transcriptome was not as robust as that of the human transcriptome. A more comprehensive view of the bacterial metabolic pathways that function *in vivo* would require higher read depth. Similarly, the differences in cell sizes and numbers between human cells and H. ducreyi hamper the ability to define active metabolic pathways by metabolomics for *in vivo* H. ducreyi; because of these differences, we assume that our metabolomics data contain little or no bacterial signal.

Our previous studies showed that ascorbic acid utilization genes in H. ducreyi and ascorbic acid were upregulated in pustules (31, 71). We confirmed those findings in this study (Fig. 5D). However, a mutant-parent human challenge study showed that an H. ducreyi mutant lacking the only known ascorbic acid transporter (*ulaABC*) in H. ducreyi was as virulent as the parent (72). This indicates that ascorbic acid is not required for bacterial growth even though it is enriched in pustules. We are unable to determine whether H. ducreyi has access to the ascorbic acid, whether ascorbic acid is sequestered in the host cells during infection, or if, in the absence of the UlaABC transporter, H. ducreyi utilizes carbon sources other than ascorbic acid during infection.

Some aspects of our data are seemingly contradictory. For example, there was upregulation of both host apoptosis genes and DNA replication genes. This is likely due to the limitations of bulk omics technologies for complex environments. For example, host cells in the abscess may be dying (e.g., neutrophils), but cells distant from the abscess (e.g., T cells, epithelial cells, and fibroblasts) may be replicating or initiating wound healing. Similarly, activation of the PPP and glycolysis is unlikely to occur in the same cell but may occur in different cells. We have also previously reported that both M1 and M2 type macrophages are present and form a collar underneath the abscess (8). Expression of *IFNG* and the presence of LOS should drive differentiation toward the M1 phenotype, while the upregulation of inflammation modulators (e.g., *ELOVL2* and *IL-10*) should drive differentiation toward the M2 phenotype. Furthermore, markers of activated M1 (*CD80* and *CD86*) as well as M2 (*MRC1*) macrophages are both upregulated in pustules (Table S3) (57). Future experiments using single-cell RNA-seq will be needed to gain higher resolution regarding the contributions of the individual cells to differential gene expression in the abscess. Understanding the spatial context of these cells will also aid in fully understanding the inflammatory environment during infection.

In summary, we successfully generated a human-H. ducreyi transcriptome interaction network and a human transcriptome-metabolome interaction network. We found that H. ducreyi was upregulating antiphagocytic effectors and microcolony adhesins *in vivo.* We observed a robust immune response composed of both pro- and anti-inflammatory genes and metabolites and a shift in multiple aspects of host metabolism. The cellular sources of these transcripts and metabolites are not currently known, but some examples of “immunometabolism” interactions seen *in vitro* are likely present here, such as metabolic flux through the PPP, the urea cycle, and fatty acid metabolism in the host. Studies that address whether the differentially regulated genes and metabolic programs can be assigned to specific cell types using single-cell RNA-seq are in progress.

## MATERIALS AND METHODS

### Bacterial strains and culture conditions.

H. ducreyi strain 35000HP was grown on chocolate agar plates supplemented with 1% IsoVitaleX in the presence of 5% CO_2_ at 33°C. H. ducreyi cells were then subcultured in GC broth supplemented with 1% IsoVitaleX, 50 μg/mL hemin, and 5% heat-inactivated fetal bovine serum and shaken at 33°C in ambient air. Mid-log-phase (optical density at 600 nm [OD_660_] of 0.200 to 0.250) cultures were processed as previously described (32). The samples used to determine H. ducreyi gene expression *in vitro* were obtained by pelleting 10 mL of mid-log-phase culture used for the human challenge experiments, removing the culture supernatant, adding 2 mL of RNAlater, incubating at room temperature for 30 min, and freezing at −80°C.

### Ethics statement.

Written informed consent was obtained from all the participants for HIV serology and for enrollment in the study. The study was approved by the Institutional Review Board of Indiana University.

### Human challenge experiments.

Dedicated stocks of strain 35000HP were used to prepare the inocula under protocols approved by the U.S. Food and Drug Administration under BB-IND no. 13064. Methods for preparation of the inoculum, inoculation of the bacteria, determination of the EDD, clinical observations, biopsy samples, and antibiotic treatment of the volunteers were performed exactly as described previously (5). Clinical endpoints included the development of a painful pustule, resolution of infection at all sites, or 14 days of observation (5). If multiple pustules formed on a volunteer, the largest pustule was biopsied.

Tissue samples used for RNA-seq were placed in 2 mL of RNAlater (Qiagen) for 30 min before freezing at −80°C; samples used for metabolomics were placed in cryotubes and flash frozen in liquid nitrogen before transfer to −80°C.

### RNA isolation and RNA-seq library preparation.

Dedicated lots of reagents and tips were used for RNA extraction of all new specimens. RNA was isolated from bacterial cultures and biopsy specimens the day after collection using the Qiagen fibrous tissue kit with the modifications described in reference 31. RNA quantity and integrity were assessed with a BioAnalyzer (Agilent).

Reagents from the Qiagen FastSelect for bacteria and for human kits were combined to deplete rRNAs from both species from the 20 RNA specimens (4 mid-log-phase cultures, 8 pustules, and 8 wounds) obtained from the 8 volunteers who were infected specifically for this study per the manufacturer’s instructions. RNA-seq libraries were prepared with a Roche KAPA kit. Libraries were pooled to account for the extra depth needed to see bacterial transcripts in the pustules. For the pilot study (31) and this study, our goal was to obtain 400 million reads from pustules, 100 million reads from wounds, and 50 million reads from mid-log cultures. Libraries were sequenced on a NovaSeq 6000 (Illumina) at the Center for Medical Genomics at Indiana University School of Medicine. Paired-end 150-bp reads were obtained. The actual numbers of reads for the new samples are presented in Table 1.

The reads obtained from the 4 volunteers who participated in the pilot study were obtained following similar procedures except the Ribo-Zero Gold rRNA removal kit (Epidemiology) (Epicentre Biotechnologies) was used to deplete human and bacterial rRNAs, libraries were constructed using the TruSeq stranded total RNA library kit (Illumina), and paired-end 75-bp reads were obtained on a Hi-Seq 4000 (Illumina).

### Differential gene expression analysis.

To compensate for the uneven numbers of human and bacterial transcripts, we subsampled the human reads to 125 million and the mid-log-phase culture reads to 1 million before alignment. All bacterial reads in the pustule and all human reads in the wound were used. Reads from the pilot study (GSE130901) were reprocessed with the same reference assemblies and annotation files. Bacterial reads were aligned against the ASM794v1 reference assembly with Bowtie2. Human reads were aligned against the hg38 reference assembly using STAR.

Bacterial and human gene count matrices were made in featureCounts. Differential gene expression was determined in DESeq2, which assumes a negative binomial distribution of the data (73). Paired analyses were performed to mitigate the influence of batch effects between this and the pilot study and to emphasize differences in gene expression due to infection rather than person-to-person variation. Clustering between sample types was assessed by principal-component analysis (PCA) of variance-stabilizing transformation (vst)-transformed data. The vegan package was used to perform a PERMANOVA for each plot. Pearson correlation coefficients were determined and plotted with the default parameters in the pheatmap package.

### Gene enrichment analyses.

To determine enriched human gene sets, DEG transcripts were presorted by descending log_2_ fold change and analyzed as a preranked list by GSEA (43). The top and bottom 20 biological processes based on normalized expression score (NES) are presented in Table 3 and Table 4, respectively. Ingenuity Pathway Analysis was used to predict activated pathways from the top 2,000 human DEGs. To determine enriched H. ducreyi genes, all H. ducreyi DEGs were sorted into categories by KEGG terms (Table 2) (33).

### Metabolite extraction.

Biopsy samples were stored at −80°C prior to shipment to the Targeted Metabolomics and Proteomics Facility at the University of Alabama at Birmingham. Each biopsy sample was first homogenized in 100 μL of PBS, and then an additional 400 μL ice-cold 80% methanol was added for 30 min to extract metabolites. The extracts were centrifuged at 3,000 × *g* for 10 min at 4°C, and the supernatant was transferred to a new test tube and dried to completion under nitrogen gas. The samples were then reconstituted in 100 μL of 0.1% formic acid in double-distilled H_2_O (ddH_2_O) for mass spectrometry evaluations.

### Untargeted metabolomics.

Sixteen tissue samples obtained from the 8 volunteers infected for this study and 6 samples from 3 volunteers who participated in the pilot study and had paired transcriptomics data (31) were analyzed for untargeted metabolomics utilizing UPLC-MS/MS and electrospray ionization quadrupole time of flight mass spectrometry (ESI Q-TOF MS). MetaboAnalyst 5.0 (74) was used for statistical evaluations. Specific chromatography and instrumentation parameters are provided in Text S1.

10.1128/mbio.03125-22.1 TEXT S1 Extended untargeted metabolomics methods. Download Text S1, DOCX file, 0.02 MB. Copyright © 2022 Brothwell et al. 2022 Brothwell et al. https://creativecommons.org/licenses/by/4.0/ This content is distributed under the terms of the Creative Commons Attribution 4.0 International license.

### Metabolomic pathway analyses.

Metabolites were classified into pathways using the Peaks2Pathways and Enrichment modules in MetaboAnalyst 5.0 (74). The Peaks2Pathways module is a wrapper for the Mummichog package (51), which uses all the MS1 spectra as the input. Pathways were assessed for significance based on a gamma distribution. Pathways containing 5 or more members where *P* is <0.05 were deemed significant. We looked for enriched pathways by MSEA using the Human Metabolite Database (HMDB) terms for the ion annotations (52). Only ions that were determined to be significantly differentially abundant by paired *t* test with Benjamini-Hochberg correction (*P* < 0.05) between the wounds and pustules were included in the enrichment analysis. Pathways containing 4 or more members were considered.

### Interaction networks.

Prior to network building, the gene counts for each sample were normalized by vst in DESeq2 and the mean centered values after normalization were calculated for each ion in MetaboAnalyst 5.0. Volunteer 467 was included in the human versus H. ducreyi transcriptome bipartite interaction network but was excluded from the metabolome versus human transcriptome interaction network as there were no matching metabolomics data for paired analysis. Only genes and ions that were determined to be differentially expressed or abundant were used in downstream network analysis.

For human and H. ducreyi transcriptome interaction networks, the log_2_ fold changes of human transcripts between infected and wounded sites and of H. ducreyi transcripts between infected sites and the inoculum used to infect the participant were calculated for each subject. The Pearson correlation coefficient between H. ducreyi and human gene expression was determined. Significant positive and negative correlations had absolute correlation coefficients of >0.75 with *P* values of <0.0005.

The xMWAS package was used for metabolome and human transcriptome interaction networks (53). A paired analysis with a PLS regression model with eigenvector centrality was used to determine correlations between individual human DEGs and the significant metabolites. Significant connections were defined as having an absolute correlation coefficient of >0.75 and *P* value of <0.05. Communities were detected using the multilevel community detection method (75). Positive and negative interactions are represented in a hybrid network plot and further annotated in Cytoscape 3.9.1.

### Data availability.

Nucleotide sequences from the pilot study and this study have been deposited in the NCBI GEO database under accession no. GSE130901 and GSE214586, respectively. Metabolomics spectra have been deposited in MassIVE under accession no. MSV000090626. All other data are available upon request from the authors.
