# Erratum for Brothwell et al., “Haemophilus ducreyi Infection Induces Oxidative Stress, Central Metabolic Changes, and a Mixed Pro- and Anti-Inflammatory Environment in the Human Host”

**DOI:** 10.1128/mbio.03522-22

**Published:** 2023-02-06

**Authors:** Julie A. Brothwell, Kate R. Fortney, Hongyu Gao, Landon S. Wilson, Caroline F. Andrews, Tuan M. Tran, Xin Hu, Teresa A. Batteiger, Stephen Barnes, Yunlong Liu, Stanley M. Spinola

## ERRATUM

Volume 13, no. 6, e03125-22, 2022, https://doi.org/10.1128/mBio.03125-22. Page 3, line 17 from bottom: “shown in Table 1” should read “shown in Table 1; the number of reads for samples from the pilot study are in reference 31.”

Page 4, Fig. 1 legend: The first sentence should read as follows. “Principal-component analysis (PCA) of the H. ducreyi transcriptomes in the inocula and pustules (A) and of the human transcriptomes in pustule and wound samples (B).”

