# Supplementary figures and images for: Haemophilus ducreyi Infection Induces Oxidative Stress, Central Metabolic Changes, and a Mixed Pro- and Anti-inflammatory Environment in the Human Host
Source: mBio. 2022 Dec 1;13(6):e03125-22. doi: 10.1128/mbio.03125-22 (PMC9765465; doi:10.1128/mbio.03125-22)

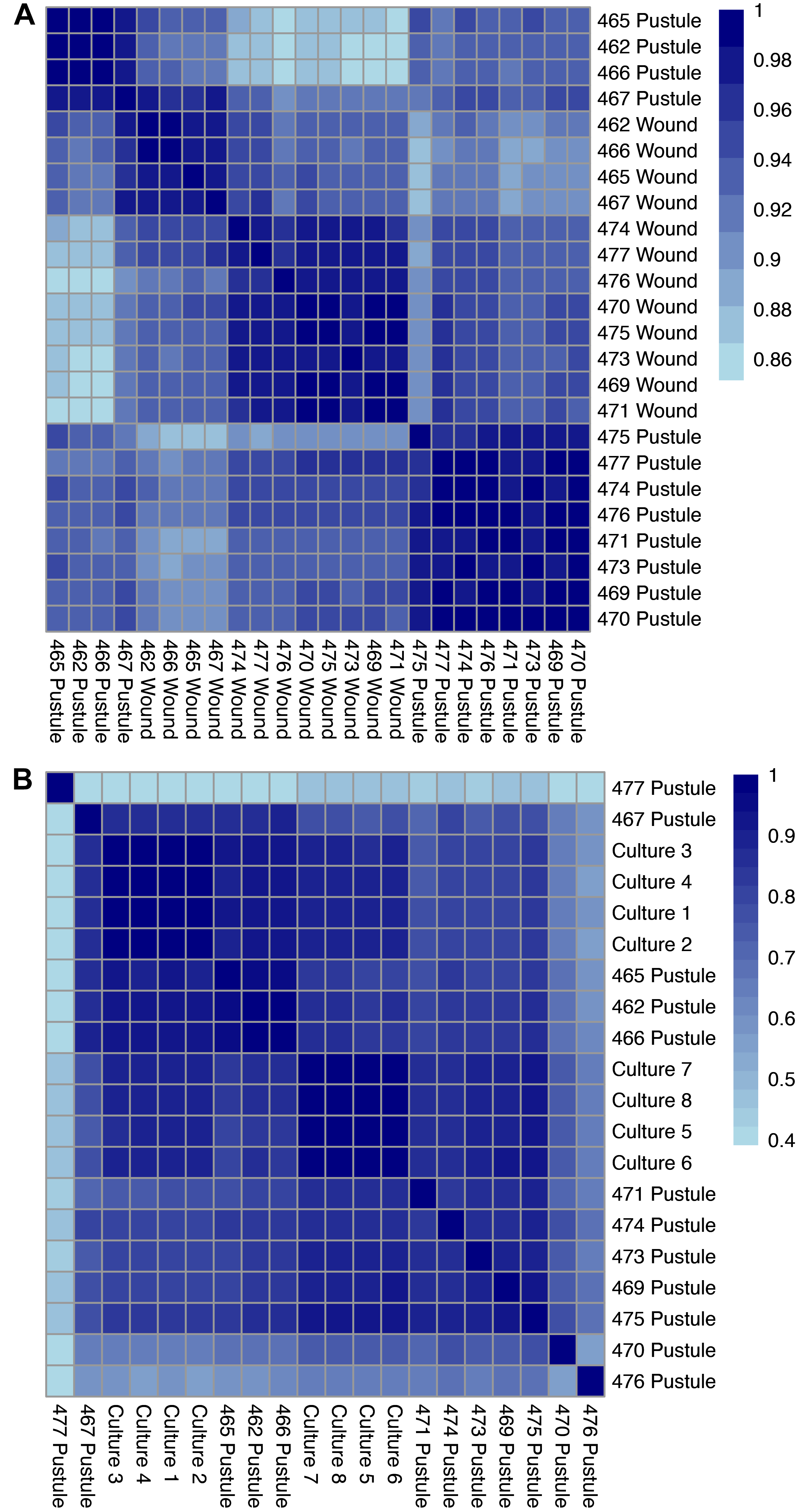

Supplement: FIG S1 [file mbio.03125-22-s0001.tif]

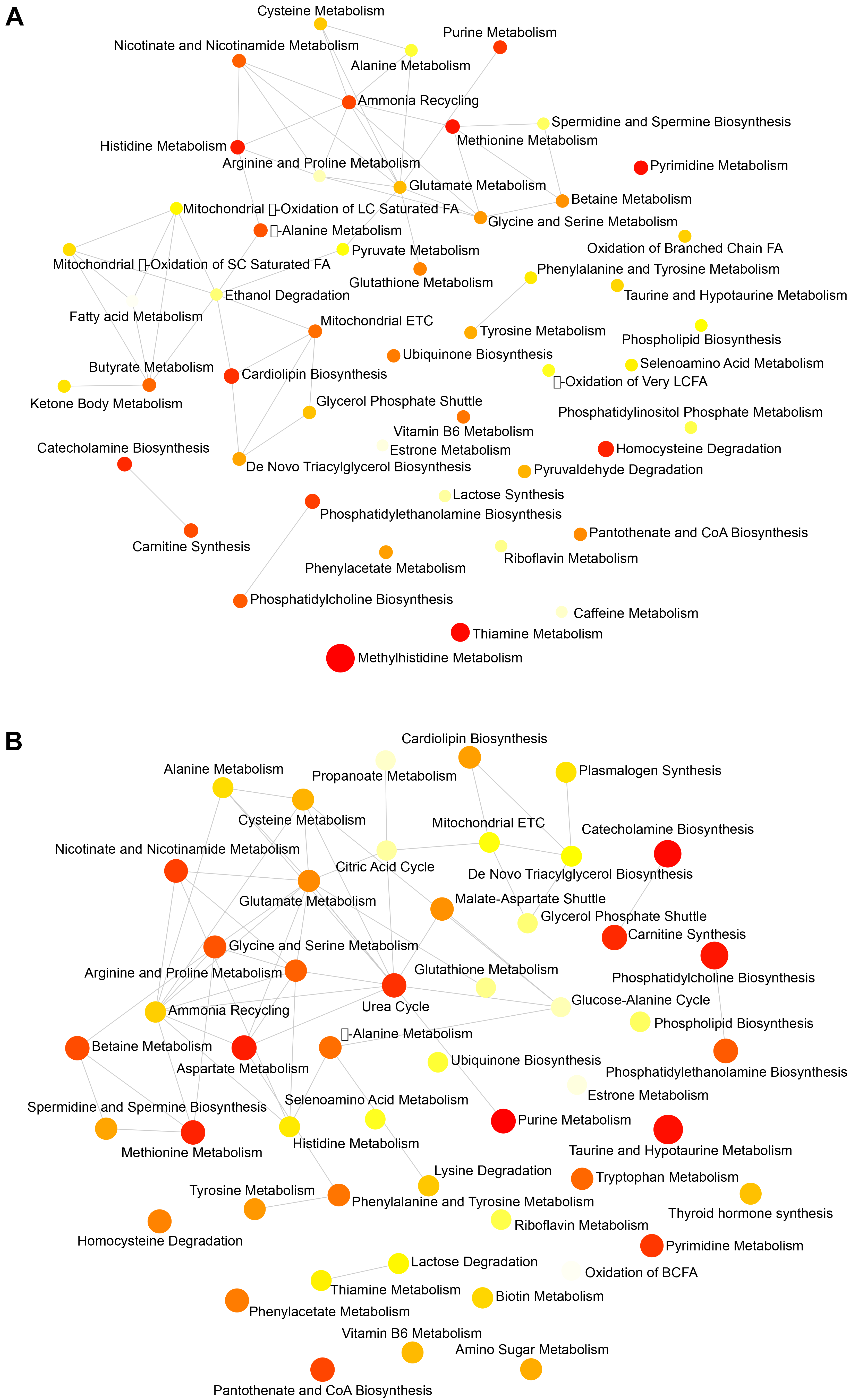

Supplement: FIG S2 [file mbio.03125-22-s0002.tif]
