# Supplementary material for: Haemophilus ducreyi Infection Induces Oxidative Stress, Central Metabolic Changes, and a Mixed Pro- and Anti-inflammatory Environment in the Human Host
Source: mBio. 2022 Dec 1;13(6):e03125-22. doi: 10.1128/mbio.03125-22 (PMC9765465; doi:10.1128/mbio.03125-22)
Supplement: TEXT S1 [file mbio.03125-22-s0003.docx]

**Supplemental Material 1: Extended Untargeted Metabolomics Methods**.

Sixteen tissue samples obtained from the 8 volunteers infected for this study and 6 samples from 3 volunteers who participated in the pilot study and had paired transcriptomics data (31) were analyzed by LC-MS/MS. An aliquot (10 μL) of each sample was loaded onto a Phenomenex 2.1 x 100mm, 2.7μm Luna Omega, 80 Å reverse-phase column (Torrance, CA). A linear gradient of 2-50% mobile phase B for 5 min, then 50-98% B until 6.0 min with a 1-minute hold, then re-equilibration at initial conditions for 3 minutes using an Exion UHPLC (Sciex, Toronto, Ontario) and a flow rate of 500μl/min. The mobile phases are A) ddH2O with 0.1% formic acid and B) acetonitrile with 0.1% formic acid respectively. The SCIEX 5600 Triple-TOF mass spectrometer (SCIEX, Toronto, Canada) was used to analyze the metabolite profile. The IonSpray voltages for positive and negative modes were +/- 5000/4500 V and the declustering potential was +/- 80 V. Ionspray GS1/GS2 and curtain gases were set at 40 psi and 25 psi, respectively. The interface heater temperature was 400°C. Eluted compounds were subjected to a time-of-flight survey scan from m/z 50-1000 to determine the top ten most intense ions for MS/MS analysis. Product ion time-of-flight scans to obtain the tandem mass spectra of the selected parent ions over the range from m/z 50-1000 were collected over 50 msec intervals using a collision energy spread of 15 eV with a set collision point of 35 eV. Spectra were centroided and de-isotoped by Analyst software, version 1.81 TF (Sciex, Toronto, Canada).

LC-MS data were processed using MS-Dial (RIKEN Center, Yokohama City, Kanagawa) to identify peaks occurring across all samples, the peak areas, and their retention times. MetaboAnalyst 5.0 (73) was used for statistical evaluations. Metabolites were identified against the IROA 600 standard compound library (IROA Technologies, Sea Girt, NJ) and verified by evaluating fragmentation spectra of each target using PeakView 2.2 software (SCIEX, Toronto, Ontario).
